# Metacognitive reflection and insight therapy: a mini-review of advances, challenges, and future directions

**DOI:** 10.3389/fpsyg.2026.1746025

**Published:** 2026-01-27

**Authors:** Courtney N. Wiesepape, Bethany L. Leonhardt, Jay A. Hamm, Laura A. Faith

**Affiliations:** 1 Department of Psychology, Indiana State University, Terre Haute, IN, United States; 2 Sandra Eskenazi Mental Health Center, Eskenazi Health, Indianapolis, IN, United States; 3 Department of Psychiatry, Indiana University School of Medicine, Indianapolis, IN, United States; 4 College of Pharmacy Practice, Purdue University, West Lafayette, IN, United States; 5 Department of Psychiatry, Richard L. Roudebush VA Medical Center, Indianapolis, IN, United States

**Keywords:** MERIT, metacognition, psychosis, psychotherapy, recovery, schizophrenia spectrum

## Abstract

Metacognitive Reflection and Insight Therapy (MERIT) is a significant development in psychotherapy for schizophrenia spectrum disorders, emphasizing meaning-making, self-understanding, and self-directed recovery. Grounded in the integrated model of metacognition, MERIT aims to help individuals form more complex and integrated understandings of themselves, others, and their community. Recent studies support its impact on metacognition, functioning, and recovery, though its flexible, process-based nature presents challenges for research standardization and widespread dissemination. This mini review summarizes MERIT’s theoretical foundations, empirical support, and clinical applications, while discussing current controversies and research gaps. We conclude by identifying future directions for expanding MERIT across diagnostic groups and furthering our understanding of metacognitive change as a mechanism of recovery.

## 1 Introduction

Recovery-oriented approaches to schizophrenia spectrum disorders (SSDs) recognize that subjective and functional recovery includes a range of meaningful improvements that extend beyond symptom reduction, such as the restoration of agency, improved self-understanding, and engagement in meaningful work, relationships, and community. Within this context, metacognition, or the capacity to identify, think about, and integrate experiences (e.g., thoughts and emotions), has emerged as a central mechanism of personal recovery (Leonhardt et al., 2019). As a result, recovery-oriented therapies increasingly target metacognitive processes to address fragmentation and support self-directed recovery.

Metacognitive Reflection and Insight Therapy (MERIT; Lysaker and Klion, 2017) is one prominent example. MERIT offers a flexible approach to promote metacognitive growth, situating therapeutic change within an individual’s developing ability to make sense of experience, relate to others, and pursue personally meaningful goals. By fostering a more integrated understanding of self, others, and the broader world, MERIT supports recovery for individuals with SSDs by helping them reestablish a sense of agency and connection, determine their purposes and possibilities, and integrate experiences into a cohesive life narrative (Faith et al., 2024).

In this review, we will present a focused synthesis of empirical and conceptual literature on MERIT, clarify theoretical debates, and highlight opportunities for future research and clinical integration. All authors are certified MERIT therapists, whose combined expertise informs the integration of foundational theory, recent empirical advances, and clinical practice. The review focuses on peer-reviewed publications from the past decade addressing the development, implementation, and evaluation of MERIT across clinical and training contexts.

## 2 Conceptual foundations

### 2.1 Theoretical framework

MERIT is rooted in the integrated model of metacognition, which conceptualizes metacognition as a spectrum of activities that allow individuals to form integrated representations of themselves and others (Moritz and Lysaker, 2018). This framework includes four domains: (1) self-reflectivity, (2) understanding the mind of the other, (3) decentration, and (4) mastery. Self-reflectivity is an individual’s ability to understand the self in increasingly complex and integrated ways. This domain ranges from recognizing discrete cognitive acts (e.g., a specific thought or emotion) to synthesizing multiple narrative episodes to form a rich, personal narrative. Similarly, understanding the mind of the other refers to the ability to form increasingly complex and integrated views of specific people, ranging from no awareness that others have thoughts and emotions to a cohesive and evolving understanding of their inner states and motivations. Decentration is the capacity to recognize that one’s broader community comprises individuals with unique lives that intersect in complex ways and that one is not the center of these interactions or events. Finally, mastery refers to the ability to use metacognitive information about the self, others, and broader world to understand and respond to psychosocial challenges. This ranges from an inability to identify plausible psychological problems to using unique metacognitive knowledge to respond to and manage psychosocial challenges that may emerge.

Importantly, it is well established that individuals with SSDs display metacognitive deficits (Lysaker et al., 2018a). For example, individuals with schizophrenia have greater deficits in metacognition when compared to other psychiatric and community samples (Lysaker et al., 2018b) and the presence of metacognitive deficits can distinguish individuals with schizophrenia from those facing other forms of medical adversity (Lysaker et al., 2014). Deficits in metacognition have also been linked to a range of outcomes, including increased difficulty managing one’s life and mental health challenges (Lysaker et al., 2018a). In contrast, intact metacognition is positively related to a broad range of psychological and social functioning, both concurrently and prospectively (Lysaker et al., 2019c). Finally, metacognitive deficits have been observed internationally and across diverse cultural contexts in individuals with SSDs (Lysaker et al., 2021), indicating that these findings are not culturally bound but a core feature.

Each domain of metacognition is a measurable construct and can be assessed using the Metacognition Assessment Scale - Abbreviated (MAS-A; Lysaker et al., 2005). Thus, the MAS-A is comprised of four corresponding subscales and a total metacognition score. These scales are scored independently, with lower scores on each scale representing greater levels of fragmentation and higher scores representing a higher degree of integration. The MAS-A has been found to have acceptable reliability and validity (Lysaker and Dimaggio, 2014). MERIT therapists use the scale to assess clients’ metacognitive functioning within session and across time to monitor progress. Using the MAS-A, MERIT therapists tailor interventions to an individual’s current metacognitive ability to scaffold higher levels of reflection through gradated practice. In sum, the MAS-A guides providers to implement interventions that target the appropriate metacognitive level and allows for measurement-based care to track progress over time.

### 2.2 Structure and process

MERIT is an integrative psychotherapy framework designed to support self-directed recovery in SSDs, offering a flexible approach that can be applied by clinicians from a wide range of theoretical and professional backgrounds (Lysaker and Klion, 2017; Lysaker et al., 2020). The acceptance of six core values has been specified as prerequisite for practicing MERIT: (1) recovery from severe mental illness is possible, (2) patients are active agents in their recovery, (3) the therapist is a consultant and equal participant, (4) experiences of psychosis can be understood, (5) greater levels of awareness may lead to emotional distress, and (6) social stigma can profoundly impact persons with SSDs.

Beyond these, every MERIT session entails eight interrelated therapist practice elements organized into three domains: content, process, and superordinate. The first four elements guide the content of sessions. The first of these is focus on the patient’s agenda, which is defined as what the patient seeks within the session and may include their hopes, desires, or needs. At any given moment, a person may have multiple agendas that can be contradictory or complementary, and these agendas may change over time (within and across sessions). Focus on the agenda helps individuals to reflect upon what they are seeking and is ultimately thought to promote agency and a shared understanding between the therapist and patient. Element 2, insertion of the therapist’s mind, encourages MERIT providers to share genuine thoughts and reactions to the patient. This establishes the clinician and patient as equal partners in dialog and promotes understanding of specific others. Element 3, eliciting narrative episodes, aims to promote the exploration of personally meaningful events within the patient’s life. A narrative episode refers to a specific situation in which individuals describe how thoughts and emotions interact to influence behavior (e.g., withdrawing at a family gathering due to self-critical beliefs and fear of embarrassment). The therapist and patient jointly reflect on these narratives, resulting in greater opportunities to integrate information about the self, others, and wider world. The final content element entails the therapist and patient collaboratively identifying a plausible psychological challenge. Of note, a plausible psychological challenge must be a subject of joint understanding and reflection. The simple acknowledgement of a diagnosis is not adequate.

The next two elements are focused on the process of therapy. Element 5 involves reflecting on the therapeutic relationship and considering the therapeutic bond and its impact on the shared work of therapy. Element 6, or reflecting on progress, includes recognizing and making sense of change within and between sessions. The final two superordinate elements involve metacognitive interventions that match a patient’s current level of metacognitive functioning. These focus on evoking and strengthening the person’s capacity for understanding self and others (Element 7) and for mastery (Element 8). Through gradated interventions, the therapist supports incremental development of metacognition over time. For example, when stimulating self-reflectivity, the therapist would avoid directly asking about or targeting emotions when a person lacks the metacognitive capacity to recognize and reflect upon those emotions. Instead, the therapist would first support the development of basic self-understanding needed for emotional awareness. Collectively, these eight elements form MERIT’s integrative and flexible structure, enabling a therapy that adapts to the individual’s evolving capacity for self-reflection, meaning-making, and connection. Table 1 provides an overview of the eight elements of MERIT.

**Table 1:** Core elements of MERIT.

Element category	MERIT therapeutic element
Content elements	Element 1: Focus on the agenda
	Element 2: Insertion of the therapist’s mind
	Element 3: Eliciting the narrative episode
	Element 4: Defining the psychological problem
Process elements	Element 5: Reflecting on the therapeutic relationship
	Element 6: Reflecting on progress
Superordinate elements	Element 7: Stimulating self-reflectivity and understanding the mind of the other
	Element 8: Stimulating mastery

## 3 Empirical support and clinical application

### 3.1 Evidence base

Empirical studies show consistent improvements in metacognition, symptoms, and functioning among individuals receiving MERIT. Both quantitative data and qualitative analyses highlight enhanced recovery after MERIT intervention. Importantly, MERIT was developed and studied in real world clinical settings and has been found to be acceptable and feasible for broad range of patients, including those with complex presentations, severe functional impairments, and some who may not historically have been offered psychotherapy (de Jong et al., 2016a; Lysaker et al., 2019a). In this regard, MERIT responds to regulatory and ethical expectations to offer recovery-oriented treatments to individuals regardless of diagnosis or clinical presentations and extends certain clinical traditions that suggested that no one was beyond the reach of therapeutic engagement (Fromm-Reichmann, 1950).

Due to MERIT’s emphasis on providing personalized interventions, it is a treatment that can be offered transdiagnostically, despite its original focus on SSDs. As such, treatment focuses less on specific diagnoses and more on understanding the person’s own view of what has gone wrong, offering interventions that match their current level of functioning, and supporting growth in their capacity to integrate information and think flexibly. This flexibility has led to explorations of MERIT in a range of populations, such as individuals experiencing first episode psychosis (MERIT-EP; Leonhardt and Vohs, 2021), while maintaining its core elements and approach.

A growing number of case studies illustrate how the flexible structure of MERIT allows for individualized adaptation and can be applied across diverse disorders and presentations (Lysaker et al., 2020). For example, case studies have demonstrated support for use with individuals with prevalent negative symptoms (van Donkersgoed et al., 2016; George and Buck, 2018), severe disorganization (de Jong et al., 2016b) and individuals diagnosed with schizoaffective disorder (Arnon-Ribenfeld et al., 2018). Clinical illustrations also demonstrate support for MERIT in clinical high risk (Leonhardt et al., 2024) and first-episode psychosis (Hillis et al., 2015; Leonhardt et al., 2016, 2018; Pattison et al., 2020) populations. Additional case studies report improved metacognition in individuals with co-occurring schizophrenia and interpersonal trauma (Hillis et al., 2018) and schizophrenia and substance use disorder (James et al., 2018). In addition, single subject studies of MERIT have expanded to other diagnostic categories. For example, case studies support the use of MERIT in the treatment of personality disorders, including borderline personality disorder (Buck et al., 2018; Vohs and Leonhardt, 2016) and schizotypal personality disorder (Cheli et al., 2019). More recently, case studies have explored application of MERIT for individuals with intellectual disability (Hamm et al., 2023a; Craig et al., 2025). Other case studies found support for MERIT’s effectiveness when delivered virtually (Faith et al., 2023).

In addition to case studies, clinical trials demonstrate support for MERIT. In one clinical trial, MERIT completers showed improved metacognition, specifically in domains of self-reflectivity and mastery, compared to treatment as usual (de Jong et al., 2019). In another trial, Vohs and colleagues found MERIT to be acceptable for individuals experiencing a first episode psychosis with treatment associated with improvements in both metacognition and clinical insight (Vohs et al., 2018). Finally, in a large randomized controlled trial of MERIT, similar improvements in self-reflectivity and mastery and reduced symptoms were found in individuals diagnosed with schizophrenia in an inpatient psychiatric ward when compared to treatment as usual (Hasson-Ohayon et al., 2024).

There is also support for MERIT from the patient’s perspective. In post-treatment interviews, MERIT completers reported experiencing therapy as beneficial for their recovery and stated that MERIT contributed to better understanding of their thinking. MERIT completers also reported that having an active role in therapy, self-expression and the therapeutic alliance contributed to positive outcomes (de Jong et al., 2020). In another study, after 12-months of MERIT, participants noted improved real-world functioning, interpersonal connections, self-compassion, and quality of life (Kukla et al., 2022).

### 3.2 Adaptations

Applications of MERIT continue to expand for use in varying disorders and presentations, including personality disorders and trauma related disorders. MERIT’s potential integration with other approaches, including compassion-focused therapy (Cheli et al., 2023) and occupational therapy approaches (Wasmuth et al., 2023), has also been explored. To date, the most robust adaptation to MERIT has been for use in group formats (MERIT-g). MERITg has been demonstrated as feasible and acceptable with outpatient and inpatient participants reporting that the group was helpful and they enjoyed hearing other perspectives (Schnakenberg Martin et al., 2024). Another study showed a significant relationship between MERITg attendance and improvement in recovery-oriented beliefs (Musket et al., 2024).

### 3.3 Mechanisms of change

Converging models indicate that metacognition partially or fully mediates the relationship between various clinical factors and functioning, with the strongest evidence for mediation between neurocognition and functioning, and growing, though more heterogeneous, evidence that metacognitive capacity helps account for the impact of symptom dimensions on everyday outcomes (Lysaker et al., 2010; Lysaker et al., 2019b). Within these models, functional capacity may represent an important process through which metacognitive abilities translate to real-world functioning. Additional recent research has explored how and to what degree different aspects of MERIT specifically relate to outcome. One session-by-session assessment found that insertion of the therapist’s mind and reflecting on progress were related to improved outcomes the following week (Lavi-Rotenberg et al., 2020). A similar session-by-session assessment found that greater emotional experience, expression, and regulation were associated with better outcomes, indicating that emotional expression is a key mechanism of change in MERIT (Igra et al., 2022). Finally, one case study of MERIT-EP found that facilitating narrative detail, interpersonal relationships, and therapist curiosity allowed for the emergence of psychological problems for joint reflection which led to improved insight (Pattison et al., 2020).

## 4 Current controversies

### 4.1 Conceptual challenges

While MERIT has gained empirical and clinical traction, several debates continue to shape its development. For example, the field continues to debate how metacognition differs from related constructs such as social cognition and mentalization. Although these constructs overlap with metacognition, important distinctions remain. Whereas social cognition and mentalization emphasize recognizing and inferring mental states in oneself or others, the integrated model of metacognition broadly focuses on the integration of these experiences into a larger, evolving sense of self, others and community. Thus, metacognition is thought to make unique contributions to understanding how an individual interprets their experiences within the flow of life. This framework also offers a description of how deficits in metacognition result in the loss of one’s sense of purpose, possibilities, and place in the world (Lysaker et al., 2021). Although initial theoretical work has begun, additional empirical studies are needed to clarify the boundaries between these constructs.

### 4.2 Implementation and dissemination

Despite growing support across various settings, populations, and geographical locations, challenges to widescale dissemination of MERIT exist, including barriers to implementation and ongoing tension between manualized and individualized treatment. MERIT is an integrative psychotherapy that intends to be flexible for both clinicians and patients and responsive to patient’s in-the-moment needs. Similarly, training clinicians in MERIT demands extensive supervision and reflection (Hamm et al., 2023b), which may be a challenge for implementation and sustainability. Additionally, few large-scale dissemination or implementation studies exist. These challenges are compounded by service systems that prioritize symptom reduction and specific functional assessments which are often at odds with MERIT’s focus on personal recovery. Likewise, various system and regulatory expectations may dictate that clinicians offer other treatments that have more widespread visibility (e.g., cognitive behavioral therapy) limiting opportunities for implementation of innovative and integrative approaches to recovery-oriented psychotherapy. While MERIT has been successfully implemented in clinical and training settings internationally, there remains a need for research evaluating strategies that consider how to balance quality training and systemic demands.

## 5 Future directions

Looking ahead, advancing MERIT will require bridging conceptual, empirical, and systemic gaps to deepen understanding of how metacognitive change supports recovery and can be sustained across diverse contexts. Future research should continue to prioritize large-scale randomized controlled trials and mechanistic studies that clarify how improvements in metacognition facilitate broader recovery and functional outcomes. Building on recent efforts to extend MERIT beyond SSDs, investigations should further examine its applicability across diagnostic groups. In turn, parallel research into the development of metacognition in normative populations could help delineate what constitutes typical versus disrupted functioning. Emerging theoretical work has also proposed metacognition as a transdiagnostic determinant of recovery (Wiesepape et al., 2024), underscoring the importance of continued integration across clinical settings and diagnoses. Finally, developing scalable training models and fidelity tools remains essential for effective dissemination and implementation of MERIT in real-world settings, efforts that are already underway.

## 6 Discussion

MERIT represents an important advancement in recovery-oriented psychotherapy, offering both a theoretical and practical framework for understanding and addressing the fragmentation of self-experience that often accompanies SSDs. While MERIT shares a focus on metacognition with related approaches (e.g., Metacognitive Interpersonal Therapy), it is distinguished by its flexible, narrative-based emphasis on supporting the integration of metacognitive capacities across self, others, and community. MERIT uses the ongoing assessment of metacognition during therapy sessions to tailor interventions that target current metacognitive capacity and promote improvement in these domains. The evidence for MERIT consistently highlights improvements in metacognition, functioning, and subjective recovery, supporting the notion that strengthening the capacity to make sense of one’s experience serves as a transdiagnostic mechanism of psychological change.

MERIT’s flexibility supports a collaborative, person-centered approach but also poses challenges for research standardization and implementation. Bridging the divide between MERIT’s phenomenological foundations and the requirements of evidence-based practice will require methodological innovation, such as session-by-session analyses that capture the process of metacognitive change. The expansion and application of MERIT to diverse clinical presentations reflects recognition that metacognitive deficits extend beyond SSDs. To sustain this growth, efforts must focus on developing scalable training and supervision models that preserve MERIT’s reflective and relational principles while ensuring fidelity, accessibility, and empirical rigor.

Looking forward, further integration between theory, research, and practice will be key to sustaining MERIT’s momentum. Continued exploration of how metacognitive growth unfolds within therapy and how this process interacts with broader systems of care can deepen our understanding of recovery itself. In this sense, MERIT’s ongoing development serves not only to refine a therapeutic model but to advance a more person- and meaning-centered vision of psychotherapy.
